# A case of splenic rupture: a rare event after laparoscopic cholecystectomy

**DOI:** 10.1186/1471-2482-14-106

**Published:** 2014-12-11

**Authors:** Girolamo Geraci, Antonino Picciurro, Andrea Attard, Giuseppe Modica, Massimo Cajozzo, Carmelo Sciumè

**Affiliations:** School of Medicine and Surgery, Section of General and Thoracic Surgery (Chief: Giuseppe Modica, MD), University Hospital of Palermo, Via Liborio Giuffrè, 5, 90127 Palermo, Sicily Italy

**Keywords:** Splenic injury, Ruptured spleen, Laparoscopic cholecystectomy

## Abstract

**Background:**

Laparoscopic cholecystectomy (LC) is generally safe and well-accepted. In rare cases, it is associated with complications (intra- e postoperative bleeding, visceral injury and surgical site infection). Splenic lesion has been reported only after direct trauma. We report an unusual case of splenic rupture presenting after “uncomplicated” LC.

**Case presentation:**

A 77-year-old woman presented with distended abdomen, tenderness in the left upper quadrant and severe anemia 12 hours after LC. Clinical examination revealed hypovolemic shock. Abdominal computed tomography confirmed the diagnosis of splenic rupture, and the patient required an urgent splenectomy through midline incision. The post-operative course was uneventful and the patient was discharged on 7th postoperative day.

Splenic injury rarely complicates LC. We postulate that congenital or post-traumatic adhesions of the parietal peritoneum to the spleen may have been stretched from the splenic capsule during pneumoperitoneum establishment, resulting in subcapsular hematoma and subsequent delayed rupture.

**Conclusions:**

Splenic rupture is an unusual but life-threatening complication of LC. Direct visualization of the spleen at the end of LC might be a useful procedure to aid early recognition and management in such cases.

## Background

Laparoscopic cholecystectomy (LC) is today the well defined “gold standard” treatment for gallstone disease, as it is well-tolerated and associated with lesser postoperative pain and discomfort, improved cosmesis, reduction of recovery and subsequent chance for early return to social activities.

However, in a small percentage of patients, LC can be complicated by bleeding, infection, bile duct injuries, retained gallstones, persistent pain, and more rarely damage to the bowel and other viscera [1].

We report an unusual case of urgent splenectomy for a ruptured subcapsular hematoma twelve hours after elective LC for cholelithiasis.

## Case presentation

A 77 years old woman with a recent history of constant right upper quadrant pain radiating to the right shoulder was referred to our hospital for symptomatic gallbladder microlithiasis. No relevant past medical history was referred and pre-operative tests did not show any pathological findings (at ultrasonography (US), spleen diameter 101 mm).

We performed a LC by 4 ports “French” technique. The pneumoperitoneum was established with open “Hasson” technique respecting an insufflating volume of 5 l/min.

We used one 10-mm trocar into umbilicus, with a 10-mm 30° laparoscope, two 5 mm trocars, respectively on the left of the midline and in the right side, and one 10 mm epigastric trocar, setting the pneumoperitoneum at 12 mmHg. No specific peritoneal adhesions around the gallbladder have been identified.

The total operative time was about 75 minutes, without any intraoperative complications or bleeding. The abdomen was normal without tenderness or guarding; no analgesic was required and there was a normal intestinal function 6 hours after surgery; normal values of postoperative (6 hours after surgery) blood tests have been found; post-operative drainage in Winslow was silent.

During the first postoperative day, at about 12 hours after the operation, she experienced self limiting lipotimic episode (pulse of 120 beats/minute, blood pressure of 80/40 mm Hg), with cold and clammy peripheries and referred a sudden upper abdominal pain. The abdominal examination showed a distended abdomen characterized by tenderness in the left upper quadrant, guarding, and rebound tenderness, Blumberg’s sign and shallow breathing.

**Figure 1:**
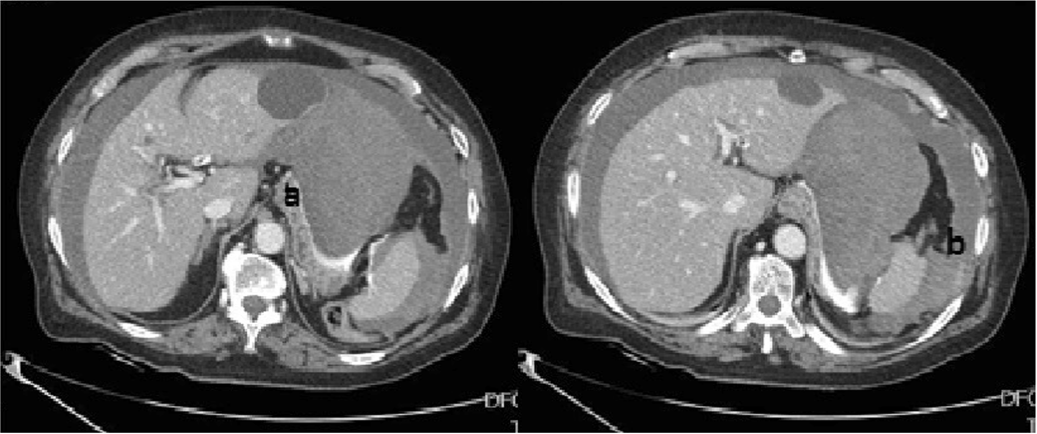
**CT showing severe haemoperitoneum with two major blood collections localized respectively along the course of hepato-gastric ligament (a) and in the left sub-phrenic space (b).**

Immediately blood tests were performed showing a severe anemia (hypovolemic shock): the Hemoglobin decreased from preoperative 10.4 gr/dl to 5.3 gr/dl and red blood cell from 3.6×10^6^/μl to 1.7×10^6^/μl. We performed an urgent computed tomography (CT) that showed severe haemoperitoneum with two major blood collections localized respectively along the course of hepato-gastric ligament (16×5 cm) and in the left sub-phrenic space (with active spreading of contrast medium) (Figure 1).

We did not consider the embolization of splenic artery before surgery because of the hemodynamic instability. The patient was urgently operated through a midline laparotomy and we found a 3-cm sub-capsular splenic haematoma ruptured into the peritoneum. We then performed splenectomy, intra-abdominal lavage (draining about 1.5 liters of blood and clots) and two drainages were placed (the first one in the splenic root and the second one in Douglas’ root). We did not perform partial splenectomy or conservation of spleen in the suspect of more complex splenic lesion.

**Figure 2:**
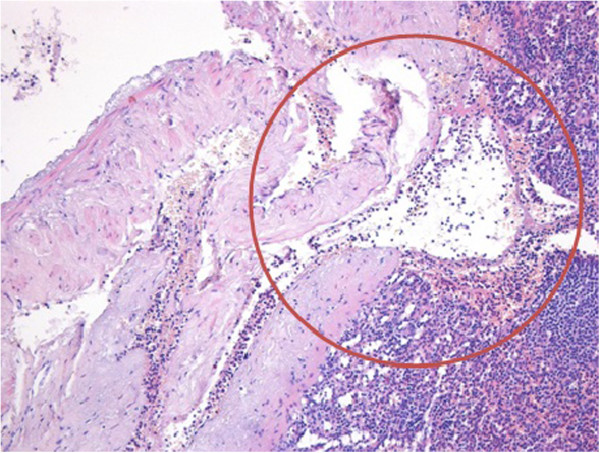
**Histological examination showing subcapsular haematoma dissecting the capsula (red circle), with normal surrounding splenic pulp.**

During the operation five blood unit transfusions, 9 plasma and 8 platelet units have been practiced. Twenty hours after the splenectomy blood tests were performed, showing a stable Hb of 9 gr/dl. No further transfusions were needed. The histological examination showed a subcapsular haematoma dissecting the capsula and rupted (in peritoneum), with normal surrounding splenic pulp (Figure 2). The remote history of the patient was negative for any kind of trauma during the preceding year, or for hematologic syndrome such as myeloproliferative or myelodisplastic disease or thrombotic thrombocytopenic purpura. The patient was discharged on the 7th postoperative day in good clinical condition, after hospitalization of 4 days in intensive care unit.

She received the standard vaccinations against encapsulated organisms (pneumococcal, meningococcal, and haemophilus influenzae).

One week after the discharge the patient performed an abdomen US scan which was negative for any intra-abdominal fluid collection.

All data reported in the manuscript have been visualized and then approved by our University Hospital Ethics Committee and all procedures carried out on the patients were in compliance with the Helsinki Declaration.

Moreover, the patient has given written explicit, express and unequivocal consent to publish her sensible data on our manuscript.

## Discussion

In a recent review, it was reported that up to 40% of all splenectomies are related to a iatrogenic splenic injury. In other studies, unplanned splenectomies range from 9% to 44% and incidental splenectomies are reported with a rate of 0.9% to 3.4% in gastric surgery, 1.2% to 8% in operations involving the left colon, 1.4% to 24% in left nephrectomies, 0.1%to 4% in abdominal vascular surgery, with an increase to 21.3% when manoeuvres of visceral rotation are performed and 60% in case of emergency surgery on the abdominal aorta. Among all abdominal operations, those performed in the upper left quadrant yield a higher rate of iatrogenic lesions (0.9% to 49%), whereas appendicectomies and cholecystectomies are the procedures with the lowest incidence of splenic injuries [1].

The splenic capsule is more frequently injured, whereas the rate of injury to the hylum and the short gastric branches of the splenic artery is lower [2].

Splenic injury following LC is more rare complication: after an extensive PubMed literature research (Search criteria: splenic, rupture, laparoscopy, lesion), we only found two manuscripts in which the patients required a splenectomy, respectively 3 weeks and 36 hours after LC (Table 1). Moreover, we also found six cases of splenic rupture during laparoscopic gynaecologic procedure (Table 2).

**Table 1:** **Splenic rupture after LC in literature**

Author	Diagnosis	CT/US	Surgery	Outcome
Leff D, JSLS 2007 [3]	Splenic rupture 3 weeks following uncomplicated LC	CT: Heterogeneous poorly enhancing soft tissue mass in the left upper quadrant inseparable from the spleen, measuring 11 x 13 cm, associated with a large volume of free intraperitoneal fluid highly suggestive of splenic rupture	Midline laparotomy with splenectomy	Discharged home one week following splenectomy
Bracale U, Ann It Chir 2013 [1]	Splenic rupture 36 hours following uncomplicated LC	US: important heamoperitoneum with two major blood collections localized respectively in the right and in the left sub-phrenic space	laparoscopic exploration + Midline laparotomy with splenectomy	Discharged home one week following splenectomy
Geraci G, 2013	Splenic rupture 12 hours following uncomplicated LC	CT: Severe heamoperitoneum with two major blood collections localized respectively along the course of hepato-gastric ligament (16 x 5 cm) and in the left sub-phrenic space (active spreading of contrast medium)	Midline laparotomy with splenectomy	Discharged home one week following splenectomy

**Table 2:** **Splenic rupture after laparoscopy**

Author	Surgery (patient)	Intraoperative	Treatment	Hypothesis
Prian DV, Am J Obstet Gynaecol, 1974 [4]	Splenic rupture after pelvic laparoscopy	Posterior subcapsularhaematoma	Midline laparotomy with splenectomy	Pneumoperitoneum stretched some adhesions between spleen, omentum and abdominal wall
Makanji HH, Br J Obstet Gynaecol, 1980 [5]	Splenic rupture after pelvic laparoscopy	Small posteriorspleniclesion	Midline laparotomy with splenectomy	Pneumoperitoneum stretched some adhesions between spleen, omentum and abdominal wall
Mahlke R, Z Gastroent 1992 [6]	Splenic rupture 5 hours following uneventful diagnostic laparoscopy (female, 48 yrs)	Small posteriorspleniclesion	Midline laparotomy with fibrin glue reparation	This complication must have occurred while establishing the pneumoperitoneum: stretching of small adhesions of the spleen with the abdominal wall may have played a role
Takeuchi K, J Reprod Med 2001 [7]	Combination of laparoscopy and preoperative trauma to the left upper quadrant of the abdomen	Sub-capsular hematoma	Midline laparotomy with splenectomy	Tearing away of delicate peritoneal reflections or small adhesions on the splenic capsule due to induction of pneumoperitoneum can result in sudden rupture and hemorrhage
Habib E, HPB (Oxford) 2004 [8]	Splenic rupture 5 days after laparoscopy for duodenal perforation closed with two sutures reinforced with fibrin glue (male, 45 yrs)	Posterior sub-capsular haematoma of the spleen at the site of the adhesion to the posterior peritoneum	Midline laparotomy with splenectomy	A posterior subcapsularhaematoma located at the site of the adhesion of the spleen to the posterior peritoneum had dissected the capsule over the dome of the spleen, and had ruptured into the peritoneum (sub capsular haematoma of the spleen that remained asymptomatic until its rupture)
Huchon C, J Min Inv Gyn 2008 [9]	Splenic rupture 5 days after diagnostic laparoscopy (female, 52 yrs with history of abdominal surgery and with acute pelvic infection)	Sub-capsular hematoma, resulting in a splenic rupture	Midline laparotomy with splenectomy	Probably caused by an overlooked puncture by the Veress needle or by tension on splenic adhesions during the adesiolysis

It is reasonable that our patient had some adhesions between the splenic capsule and the parietal peritoneum. So, when the pneumoperitoneum was estabilished at the start of the LC, stretching of the splenic capsule resulted in a small sub-capsular hematoma. This also seems to be supported by our histological findings. In addiction, the temporal proximity between LC and splenectomy, the lacking of history of abdominal trauma and the histological absence of splenic intrinsic pathological abnormality, confirm that splenic rupture represents a primary complication of LC [3].

In the present case, haemoperitoneum and bleeding shock appeared about 12 hours following LC, when the subcapsular haematoma ruptured into peritoneum.

About the use of drainage during an uncomplicated LC, we follow the recommendation of a recent Cochrane Review in which “the Authors could not find evidence to support the use of routinary drainage after LC” [10].

## Conclusions

Splenic rupture is an unusual but serious complication of LC. It has to be diagnosed and treated if possible during the laparoscopic procedure. The identification of a lesion of the anterior edge or the inferior pole of the spleen is possible during laparoscopy if the spleen is observed at the end of the procedure, whereas the detection of a posterior subcapsular haematoma of the spleen is impossible during laparoscopy and cannot be suspected if there is no bleeding from the spleen at the end of the procedure. Such a haematoma is not detectable after laparoscopy if the patient remains asymptomatic, and symptoms only occur after the haematoma ruptures.

However, we suggest visualization of the spleen at the end of LC to ensure early recognition and management of such cases. Obviously, manipulating the spleen is not necessary but we do believe it would be reasonable to wait for some seconds and see if there is any bleeding.

Imaging may be useful to diagnose such a haematoma but ultrasound and CT are unlikely to be performed in asymptomatic patients.
